# Use of antiemetics in children with acute gastroenteritis: Are they safe and effective?

**DOI:** 10.4103/0974-2700.44674

**Published:** 2009

**Authors:** Jacob Manteuffel

**Affiliations:** Henry Ford Hospital, 2799 W. Grand Blvd, Detroit, MI 48201, USA

**Keywords:** Antiemetics, gastroenteritis, pediatrics

## Abstract

The use of antiemetics is a controversial topic in treatment of pediatric gastroenteritis. Although not recommended by the American Academy of Pediatrics, antiemetics are commonly prescribed by physicians. A review of the literature shows side effects of promethazine, prochlorperazine, and metoclopramide are common and potentially dangerous. Ondansetron has recently been studied as an adjunct to oral rehydration therapy in treatment of acute gastroenteritis with mild to moderate dehydration. Although studies are limited, early research suggests the medication is safe when used in a single dose and can be effective to prevent vomiting, the need for intravenous fluids, and hospital admission.

A 2-year-old female presents to the emergency department (ED) from family practice clinic with a 2 day history of vomiting, diarrhea, and a failed attempt at oral rehydration in the clinic. The mother states she herself recently got over the “stomach flu.” She has had six wet diapers today. On physical examination, she is non-toxic, appearing restless in her mothers arms, crying with tears, and has vital signs within normal limits for her age. Her lips appear dry, but her tongue is moist and her abdomen is soft and non-tender. The working diagnosis is acute gastroenteritis with mild dehydration and you explain the process of oral rehydration to the mother and give reassurance. The mother is hesitant to take her child home because she vomited in the clinic and that is why she was sent to the ED. The patient is given 50 ml of an oral rehydration solution to drink slowly, which she keeps down, the nurse brings the patient's discharge paperwork and the girl vomits again. Should the ED physician continue with plans for discharge? Should the physician order a bolus of intravenous normal saline and/or consider admission? Are there any medications that may aid in oral rehydration that are safe and effective?

## EPIDEMIOLOGY

Acute gastroenteritis (AGE) in the pediatric population is a common problem in the emergency department and accounts for at least 1.5 million visits to primary care providers each year in the United States. It accounts for approximately 10% of all hospital admissions for children under the age of 5 and approximately 220,000 hospitalizations each year. The cost of each admission is approximately $1900. Four hundred deaths per year are attributed to the dehydration caused by gastroenteritis. Over 60% of cases of AGE which present to the ED are of viral cause, with the most common being rotavirus.[1,2]

## PATHOPHYSIOLOGY

The mechanism of vomiting in gastroenteritis is not completely understood. One of the proposed mechanisms is thought to be initiated by serotonin stimulation of 5HT-3 receptors in the stomach and small intestine as well as the vagus nerve. These receptors send afferent nerve impulses to the chemoreceptor trigger zone (CTZ) and the vomiting center (VC) in the brain stem which cause the diaphragm, abdominal muscles, and visceral smooth muscle to produce vomiting.[3]

## DEHYDRATION AND ORAL REHYDRATION THERAPY

Children are more susceptible to the effects of fluid loss and electrolyte abnormalities because of physical size. There is no widely accepted classification system for dehydration in children.[1] Most physicians use clinical judgment based on a series of physical exam findings to determine the severity of dehydration[4] [Table 1]. Oral rehydration therapy (ORT) as determined by the World Health Organization (WHO) has been shown to be safe and effective for fluid repletion in infants and children with AGE and mild to moderate volume depletion. ORT can be instituted if the patient continues to vomit or have diarrhea. There are a number of oral rehydration solutions (ORS) to choose from, the most common of which is Pedialyte, (Abbot Nutrition, Columbus OH) which is slightly hypotonic to intravascular fluid. The protocol for ORT is to establish the degree of dehydration, and use 50 ml/kg of ORS for mildly dehydrated children and 100 ml/kg for moderately dehydrated children. Twenty five percent of the established volume is administered each hour for a four-hour period. If the patient fails this therapy intravenous fluids (IVF) are indicated.[5]

**Table 1:** Example physical exam findings in dehydration[4]

Variable	Normal or mild dehydration	Moderate dehydration	Severe dehydration
Pinch retraction time	Immediate	Slow (<2 sec)	Very slow (>2 sec)
Feeling of skin to touch	Normal	Dry	Clammy or cool
Condition of buccal mucosa	Moist	Dry	Very dry
Heart rate	Within normal limits	Tachycardia (<10% above normal)	Tachycardia (>10% above normal)
Urine	Normal amount and color	Reduced amount or darker in color	None passed in >6 h
Tears	Present	Reduced	None
Mental status	Thirsty, alert	Drowsy, irritable, restless	Limp, lethargic

COPYRIGHT 2006 *MASSACHUSETTS MEDICAL SOCIETY. ALL RIGHTS RESERVED.*

## AAP GUIDELINES

The American Academy of Pediatrics (AAP) recommends ORT as the treatment of choice in the mild to moderate dehydration and is as effective as IV therapy. They recommend starting an age-appropriate diet as soon as the patient is rehydrated. The routine use of anti-diarrhea agents is not recommended because of potential side effects. There is no mention of antiemetic use in their guidelines.[5]

## CLINICAL PRACTICE

Review of the literature shows clinicians commonly use and prescribe antiemetics for vomiting in children with AGE. A retrospective study of 20,000 children with AGE showed 9% of patients had a prescription filled for an antiemetic. In addition, 5% of patients under the age of 2 had a prescription filled for an antiemetic, the most common of which was promethazine (Phenergan).[6] A survey of Italian pediatricians reported a 79% use of antiemetics for AGE.[7] A survey of emergency medicine (EM), Pediatrics, and Pediatrics/EM boarded physicians reported a 61% use of antiemetics at least once in the past year. Promethazine per rectum was the most common medication used.[2]

## MEDICATIONS

Promethazine is a H1 receptor antihistamine which inhibits the VC from peripheral stimulants.[2] The most common side effect of promethazine is respiratory depression and sedation which caused the FDA in 2004 to issue a “boxed warning” contraindicating the medication for children less than 2 years old.[8] Numerous case reports detail other extra pyramidal side effects including torticollis in therapeutic doses of promethazine.[9] Another study showed a higher incidence of promethazine use in SIDS related cases.[10]

Prochlorperazine (Compazine) was first introduced as an anti-psychotic in the 1950s, and subsequently found to be effective to control vomiting. It is a weak dopamine receptor blocker and depresses the CTZ.[2] Akathisia and dystonia are the most common reported side effects in adults and children in up to 44% of patients administered this medication.[2,11–13]

Metoclopramide (Reglan) is a dopamine receptor antagonist which acts both centrally and peripherally, increases gastric motility and decreasing afferent impulses to the CTZ. A review of the pediatric literature reports akathisia and dystonia in up to 25% of children receiving this medication.[2]

## RECENT RESEARCH

Ondansetron (Zofran) has been proven safe and effective in chemotherapy induced and post operative vomiting. It is a selective serotonin 5HT-3 receptor blocker and inhibits the initiation of the vomiting reflex in the periphery. In 1997, Cubeddu was the first to demonstrate the antiemetic effects of ondansetron in AGE.[3] Reeves, 2002, also demonstrated the antiemetic properties of ondansetron and a decreased hospital admission rate in those with a serum CO_2_ >15 mEq/L.[14] Ramsook, 2002, was the first to compare oral ondansetron to placebo again demonstrating its antiemetic effect and also a decreased need for IVF and hospital admission. Significantly higher rates of diarrhea were reported in this study related to ondansetron as additional doses of this medication were given at discharge.[15] In 2006, Freedman published a study in the New England Journal of Medicine (NEJM) demonstrating the antiemetic properties of oral ondansetron with a number needed to treat (NNT) of 5 to prevent vomiting and a NNT of 6 to prevent the need for IVF. This medication was given as a single dose only[4] [Table 2].

**Table 2:** Outcome measures from Freedman study[4]

Outcome	Ondansetron group (*N*=107)	Placebo group (*N*=107)	Relative risk (95% CI)	*P* value
Vomited during oral rehydration - no. (%)	15 (14)	37 (35)	0.40 (0.26-0.61)	<0.001
Mean no. of vomiting episodes	0.18	0.65	0.30 (0.18-0.50)	<0.001
Vomiting episodes per patient - no. (%)					
0	92 (86)	70 (65)		<0.001
1	12 (11)	21 (20)		0.13
2	2 (2)	7 (7)		0.17
>2	1 (1)	9 (8)		0.02
Intravenous rehydration - no. (%)	15 (14)	33 (31)	0.46 (0.26-0.79)	0.003
Hospitalization - no. (%)	4 (4)	5 (5)	0.80 (0.22-2.90)	1.00
Oral-rehydration fluid consumed (ml)	239±112	196±92		0.001
Intravenous fluid administered (ml/kg)	38±8.9	46±9.1		0.002
Length of time of stay in emergency department (min)	106±53	120±63		0.02

COPYRIGHT 2006 *MASSACHUSETTS MEDICAL SOCIETY. ALL RIGHTS RESERVED.*

Roslund, 2008, demonstrated an improved success rate of ORT, a decreased need for IVF, and a decreased hospitalization rate in patients with AGE treated with a single dose of oral ondansetron, who initially failed ORT in the ED. Rates of diarrhea on follow up were similar to placebo.[16] This study again suggests when ondansetron is used in a single dose there appear to be no side effects.

## SUMMARY

Prochlorperazine, promethazine, and metoclopramide have a high incidence of side effects and should be avoided in patients less than 2 years old and used with extreme caution in children older than 2 years.[2,8–13] In limited studies, ondansetron when used as a single dose has shown to be safe in children with acute gastroenteritis. In addition, ondansetron has recently become generic and cost is no longer a barrier to routine use in the ED. Oral ondansetron could be a consideration for children with AGE who fail ORT to prevent the need for IVF, or as an adjunct to IVF to help facilitate ORT and prevent admission. The dose of this medication can be weight based, approximately 0.1 mg/kg.[4,16] If an ED physician decides to use ondansetron it should only be given as a single dose in the ED, as further doses have shown to cause persistent diarrhea.[15] Presently in the literature there are five studies which examine the safety and effectiveness of ondansetron in pediatric patients with AGE.[3–4,14–16] Therefore, further studies and a subsequent meta-analysis are required to determine whether ondansetron is an effective adjunct therapy to ORT in AGE.

## RESOLUTION OF CASE

In the case of the 2 year old with vomiting and diarrhea, the girl was given a 2 mg ondansetron oral dissolving tablet and observed in the emergency department for 2 h. She was able to tolerate 10 ml/kg of pedialyte every 10 min without vomiting. At this point, the patient was discharged with her mother to continue ORT for 2 h, then to resume regular diet.
